# Supplementary material for: HIVprotI: an integrated web based platform for prediction and design of HIV proteins inhibitors
Source: J Cheminform. 2018 Mar 9;10:12. doi: 10.1186/s13321-018-0266-y (PMC5845081; doi:10.1186/s13321-018-0266-y)
Supplement: Supplementary file 2 — Additional file 2. Source code of HIVProtI web server. [file 13321_2018_266_MOESM2_ESM.zip › HIVprotI_Source-code/hivprotI/weka-3-6-6/doc/weka/gui/beans/Visible.html]

Visible


---


|  |  |  |
| --- | --- | --- |
| **Overview** \| **Package** \| **Class** \| **Tree** \| **Deprecated** \| **Index** \| **Help** \| **Weka's home** | |  |
| **PREV CLASS**   **NEXT CLASS** | **FRAMES**    **NO FRAMES**     **All Classes** |  |
| SUMMARY: NESTED \| FIELD \| CONSTR \| METHOD | DETAIL: FIELD \| CONSTR \| METHOD |  |


---


## weka.gui.beans Interface Visible

**All Known Implementing Classes:**: AbstractDataSink, AbstractDataSource, AbstractEvaluator, AbstractTestSetProducer, AbstractTrainAndTestSetProducer, AbstractTrainingSetProducer, Associator, AttributeSummarizer, ClassAssigner, Classifier, ClassifierPerformanceEvaluator, ClassValuePicker, Clusterer, ClustererPerformanceEvaluator, CostBenefitAnalysis, CrossValidationFoldMaker, DataVisualizer, Filter, GraphViewer, IncrementalClassifierEvaluator, InstanceStreamToBatchMaker, Loader, MetaBean, ModelPerformanceChart, PredictionAppender, Saver, ScatterPlotMatrix, SerializedModelSaver, StripChart, TestSetMaker, TextViewer, TrainingSetMaker, TrainTestSplitMaker

---

``` public interface Visible ```

Interface to something that has a visible (via BeanVisual) reprentation

**Since:**
:   1.0

**Version:**
:   $Revision: 1.2 $

**Author:**
:   Mark Hall

---

| **Method Summary** | |
| --- | --- |
| `BeanVisual` | `getVisual()`             Get the visual representation |
| `void` | `setVisual(BeanVisual newVisual)`             Set a new visual representation |
| `void` | `useDefaultVisual()`             Use the default visual representation |

**Method Detail**

### useDefaultVisual

```
void useDefaultVisual()
```

:   Use the default visual representation

---


### setVisual

```
void setVisual(BeanVisual newVisual)
```

:   Set a new visual representation

    :   **Parameters:**: `newVisual` - a `BeanVisual` value

---


### getVisual

```
BeanVisual getVisual()
```

:   Get the visual representation

    :   **Returns:**: a `BeanVisual` value


---


|  |  |  |
| --- | --- | --- |
| **Overview** \| **Package** \| **Class** \| **Tree** \| **Deprecated** \| **Index** \| **Help** \| **Weka's home** | |  |
| **PREV CLASS**   **NEXT CLASS** | **FRAMES**    **NO FRAMES**     **All Classes** |  |
| SUMMARY: NESTED \| FIELD \| CONSTR \| METHOD | DETAIL: FIELD \| CONSTR \| METHOD |  |


---
